# MaABI5 and MaABF1 transcription factors regulate the expression of *MaJOINTLESS* during fruit abscission in mulberry (*Morus alba* L.)

**DOI:** 10.3389/fpls.2023.1229811

**Published:** 2023-08-21

**Authors:** Xuan Deng, Bilal Ahmad, Jing Deng, Lianlian Liu, Xiuping Lu, Zelin Fan, Xingfu Zha, Yu Pan

**Affiliations:** ^1^ State Key Laboratory of Resource Insects, Southwest University, Chongqing, China; ^2^ State Key Laboratory of Tropical Crop Breeding, Ministry of Agriculture and Rural Affairs, Agricultural Genomics Institute at Shenzhen, Chinese Academy of Agricultural Sciences, Shenzhen, China; ^3^ Guangdong Laboratory of Lingnan Modern Agriculture, Ministry of Agriculture and Rural Affairs, Agricultural Genomics Institute at Shenzhen, Chinese Academy of Agricultural Sciences, Shenzhen, China; ^4^ Key Laboratory of Synthetic Biology, Ministry of Agriculture and Rural Affairs, Agricultural Genomics Institute at Shenzhen, Chinese Academy of Agricultural Sciences, Shenzhen, China; ^5^ College of Horticulture and Landscape Architecture, Southwest University, Chongqing, China

**Keywords:** *Morus alba L.*, fruit abscission, *MaJOINTLESS*, transcriptome, ABA (abscisic acid)

## Abstract

Mulberry holds significant economic value. However, during the ripening stage of its fruit, the phenomenon of abscission, resulting in heavy fruit drop, can severely impact the yield. The formation of off-zone structures is a critical factor in the fruit abscission process, and this process is regulated by multiple transcription factors. One such key gene that plays a significant role in the development of the off-zone in the model plant tomato is *JOINTLESS*, which promotes the expression of abscission-related genes and regulates the differentiation of abscission zone tissue cells. However, there is a lack of information about fruit abscission mechanism in mulberry. Here, we analyzed the *MaJOINTLESS* promoter and identified the upstream regulators MaABF1 and MaABI5. These two regulators showed binding with *MaJOINTLESS* promoter MaABF1 (the ABA Binding Factor/ABA-Responsive Element Binding Proteins) activated the expression of *MaJOINTLESS*, while MaABI5 (ABSCISIC ACID-INSENSITIVE 5) inhibited the expression of *MaJOINTLESS*. Finally, the differentially expressed genes (DEGs) were analyzed by transcriptome sequencing to investigate the expression and synergistic relationship of endogenous genes in mulberry during abscission. GO classification and KEGG pathway enrichment analysis showed that most of the DEGs were concentrated in MAPK signaling pathway, flavonoid biosynthesis, citric acid cycle, phytohormone signaling, amino acid biosynthesis, and glycolysis. These results provide a theoretical basis for subsequent in-depth study of physiological fruit abscission in mulberry.

## 1 Introduction

Fruit abscission of fruit trees is a common phenomenon that occurs in plants and is regulated by internal and external factors. There are two types of fruit abscission: physiological fruit abscission and abnormal fruit drop. Physiological fruit abscission is a normal phenomenon that occurs due to internal factors during the normal growth and development of the fruit tree. Abnormal fruit drop, on the other hand, is caused by abnormal environmental climates, insufficient nutrition, biological stress, and other factors during fruit development. To a certain extent, fruit abscission can limit the fruit yield of fruit trees and affect the industrial harvest of fruits. Mulberry, in particular, is known to be more susceptible to fruit drop during the ripening process.

Mulberry is an important woody perennial plant and has multiple benefits. For example, mulberry leaves can be used for rearing silkworms, and its fruits are even more popular and contain a large number of nutritional compounds. Such as fatty acids, amino acids, vitamins, minerals, and bioactive substances. The extracts and active ingredients of mulberry fruit also have important medicinal values, such as antioxidant, immunomodulatory and antitumor effects (Yuan and Zhao, 2017). In mulberry there is a problem of heavy fruit drop at the fruit ripening stage, which leads to less yield. Therefore, we aimed to investigate the molecular mechanism of fruit drop in mulberry for investigating the theoretical basis.

Abscission occurs in a specific region of the cell called the abscission zone (AZ), which consists of several layers of dense cytoplasmic cells. Initially, researchers reported that the complete abscission process has three stages; the signaling phase, the regulation stage, and the execution phase (Marcelis et al., 2004). However recent studies suggest that the abscission process has four stages. In the signaling phase, the action of developmental signals (pollination, senescence, fruit ripening, etc.) and environmental signals (light, temperature, water, etc.) allow the gradual formation of AZ (Marcelis et al., 2004). In the regulation stage, the AZ can perceive different stimuli generated by external and internal factors and, through signaling, activates the signaling response of ethylene or abscisic acid (ABA). In the execution phase, shedding starts with the expression of cell wall hydrolases, such as cellulases, polygalacturonases (Kim, 2014). The collective action of all these substances accelerates the lysis of the intermediate lamellae and finally, cell wall lysis occurs, leading to the separation of cells or organs. In 2018, a physical model of fruit abscission was developed, in which abscission is divided into four stages. In the first stage, the AZ is formed under the action of several transcription factors. In the second stage, the abscission signal is activated. In the third stage, enzymatic hydrolysis of the middle thin layer of AZ occurs, and polygalacturonases (PGs), pectin methyltransferases (PMEs), and expansion proteins (EXP) lead to cell separation. Finally, the abscission scar further differentiates and closes itself (Kim, 2014).

Plant hormones play an important role in the process of fruit abscission. ABA has been reported to regulate plant growth and development, stress responses, an physiological processes such as stomatal closure, leaf senescence, shoot dormancy, seed germination, osmoregulation, and growth inhibition (Chen et al., 2020). Ethylene (ETH) is a gaseous plant hormone that promotes fruit ripening, senescence, and abscission (Merelo et al., 2017). ETH response factors (AP2/ERF) are a class of transcription factors that regulate the expression of ethylene-responsive genes and are involved in fruit ripening, morphological changes, signal transduction, and organ senescence (Feng et al., 2020). Auxin plays a very important role in plant growth and development. Auxin response factor (ARF) is a family of transcription factors that regulate the expression of auxin-responsive genes (Tiwari et al., 2003). Studies have shown that ARF is important in plant growth and development such as flower and cotyledon development, plant embryogenesis, senescence of leaf organs, and fruit ripening (Tiwari et al., 2003). The direct cause of fruit abscission is the degradation of the cell wall, which is closely related to cellulase (EG), pectinase, peroxidase (POD) (Dietz, 2003), polygalacturonase (PG) (Bonghi et al., 1992), and expansins (EXP) (Li et al., 2003). In addition, carbohydrates can provide nutrients for fruit development, and when carbohydrates are insufficient, they may cause nutrient stress and result in fruit abscission. It has been shown that sugar signaling can induce ABA and ETH synthesis to increase reactive oxygen species (ROS) content and generate abscission signals, which are transmitted to the AZ and lead to fruit abscission (Sawicki et al., 2015). Moreover, carbohydrate stress has been shown to cause fruit abscission in litchi (Kuang et al., 2012).

In plants, abscission is a common phenomenon like leaves, flowers, and fruits shedding. The abscission of plant organs cannot be separated from the development of theAZ. The *JOINTLESS* gene has been identified in tomato and is able to regulate the development of the free zone of the tomato floral organ (Mao et al., 2000). A mutation in the *JOINTLESS* gene resulted in the absence of detached tissue in mutant tomatoes, while wild-type tomatoes showed detached tissue, as evidenced by staining longitudinal sections of pedicels with methanetriol (Roldan et al., 2017). Further, the MADS-box family gene MACROCALYX (MC) can affect the development of tomato sepals and inflorescences (Vrebalov et al., 2002). MC and JOINTLESS interact to regulate the development of AZ in tomato fruits (Nakano et al., 2012). The knockdown of the SEPALLATA (SEP) MADS-box protein SLMBP21 eliminated flower AZ development and SLMBP21 formed a protein complex with JOINTLESS and MC as a transcriptional activator of tomato flower AZ development (Liu et al., 2014). In 2017, scientists cloned the *PsJOINTLESS* gene in Kuril balsam pear, and after the overexpression of *PsJOINTLESS* gene in tomato resulted in increased pedicel abscission rate more AZ cell layers, thinner pedicels, smaller pedicel cells, and higher pedicel AZ cellulase activity. These findings confirm the involvement of *PsJOINTLESS* in pedicel AZ development (Qi et al., 2018). In addition, there are many transcription factors associated with the development of the isolated region, such as WUSCHEL (WUS), BLIND (Bl), GOBLET (GOB), and LATERAL SUPRESSOR (Ls), and BLADE-ON-PETIOLE (BOP) (Nakano et al., 2012). Moreover, the tomato lateral repressor gene Ls encodes the VHIID protein family, which is involved in the development of AZ (Schumacher et al., 1999). *Arabidopsis BOP1* and *BOP2* promote asymmetric flower and leaf morphology, and mutants of both exhibit floral organ abscission (McKim et al., 2008). The homologous gene *NbBOP2* was found to mediate the differentiation of the deciduous zone of the corolla in tobacco (Wu et al., 2012). The development of the off-zone is closely related to the abscission of fruits.

At present, there are few studies related to mulberry fruit shedding. There is lack of information about the mechanism of higher fruit shedding in mulberry at ripening stage, our study will explore molecular basis and will provide basis for solving the issue.

## 2 Materials and methods

### 2.1 Plant materials and growth conditions

The mulberry (*Morus alba* L., ‘Hongguo 2 hao ‘) and tobacco seeds (*Nicotiana benthamiana*) were taken from the State Key Laboratory of Silkworm Genome Biology, Southwest University, Chongqing, China. Tobacco and mulberry seeds were grown in a mixture of vermiculite and nutritive soil (1:1) at 25°C, 40% to 60% relative humidity, under a 16h light/8h dark photoperiod. Tobacco plants were used for transient expression assays.

In June 2021, samples for transcriptome sequencing were gathered from the experimental site of the State Key Laboratory of Silkworm Genome Biology at Southwest University. Only mature and healthy mulberry fruit stalks were chosen, and a total of 12 fruit stalks were collected as a sample. To ensure accuracy, three biological replicates were used. The detached area tissues of fruit stalks that are easy to drop fruits were taken as the experimental group, marked as MD_ 1, MD_ 2, and MD_ 3. The tissues of fruit stalks that were not easy to drop fruits were taken as the control group, marked as MN_ 1, MN_ 2, and MN_ 3. After quick freezing in liquid nitrogen, samples were stored in an ultra-low temperature freezer (- 80°C).

### 2.2 Cloning of *JOINTLESS* promoter and histochemical GUS assays

**Figure 1:**
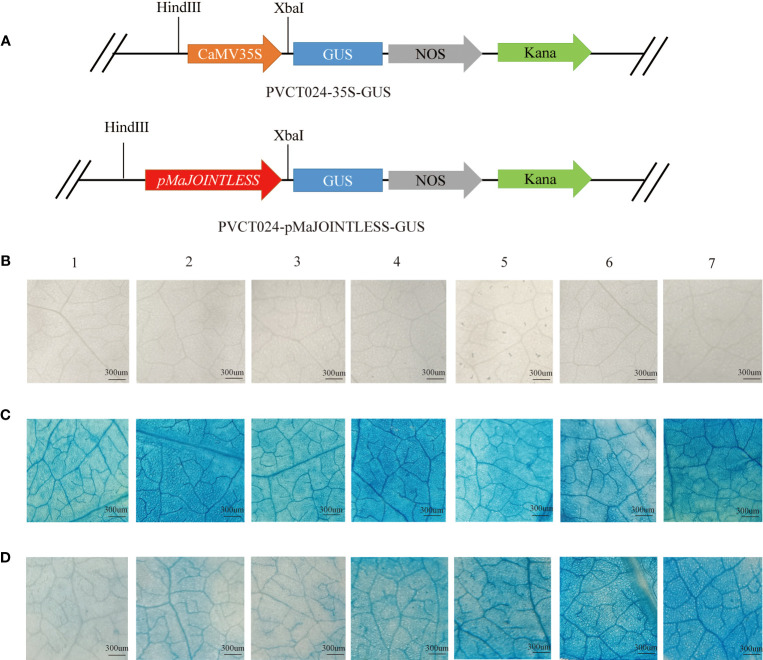
GUS expression driven by the *MaJOINTLESS* promoters in *Nicotiana benthamiana.* **(A)** Schematic diagram of carrier construction of *pMaJOINTLESS*-PVCT024. **(B)** Negative control group. **(C)** Positive control group, *35S: GUS*, GUS expression driven by the 35S promoters in the leaves. **(D)** Experimental group, *proMaJONTLESS: GUS*, GUS expression driven by the *MaJOINTLESS* promoters in the leaves. 1-7 represent different stress treatments.1: Dark. 2: Light. 3: Water. 4: 2,4-D. 5: GA3. 6: Ethephon. 7: ABA. Scale bars are 300 μm.

The *MaJOINTLESS* promoter sequence was predicted by local blast from the genome file of mulberry, using the first base of the *MaJOINTLESS* promoter sequence as +1 and the first base upstream of ATG as -1. Further, 1500 bp upstream promoter sequence was cloned and named *pMaJOINTLESS*. The extraction of genomic DNA was performed using Plant Genomic DNA Extraction Kit (DP3111, BioTeke, Beijing, China) according to the instructions. The promoter fragments of *MaJOINTLESS* (1500 bp) were subcloned into the PVCT024 vector upstream of the GUS (β-glucuronidase)-coding region (**Figure 1A**). The construct was transformed into tobacco leaves. The leaves were incubated in GUS staining buffer (0.2 mL 0.5 M EDTA, 0.3 mL 40 mM X-Gluc, 0.1 mL 10% TritonX-100, 1 mL 1 M Sodium hydrogen phosphate buffer) for 3 h at 37°C in the dark. The samples were then decolorized in 100% ethanol and visualized using a Zeiss SV11 stereoscope (Carl Zeiss Jena, Oberkochen, German).

### 2.3 Sequence analysis and quantitative real-time PCR

Protein sequences of all candidate genes were retrieved from the *Arabidopsis* genome database (https://www.arabidopsis.org/), and the tobacco genome database (https://solgenomics.net/organism/Nicotiana_benthamiana/genome). Multiple alignments were performed using the DNAMAN. Total RNA was extracted with TRIzol^®^ reagent (Invitrogen, Carlsbad, CA, USA) according to the manufacturer’s protocol. The optical density (OD) value of RNA was measured by spectrophotometer, and the total RNA with the purity (260/280) of 1.9 to 2.0 was used for further assay. Total RNA template was reverse transcribed into cDNA with a PrimeScript RT reagent Kit with gDNA Eraser (TaKaRa, Shiga, Japan). The synthesized cDNA was diluted to 200 ng/µL as the template for qPCR. qPCR experiments were carried out on ABI7500 Real-Time PCR machine (Applied Biosystems, Foster City, CA, USA). Then qPCR was performed to quantify the RNA levels with NovoStart^®^ SYBR qPCR SuperMix Plus (Novoprotein, Shanghai, China). All procedures with instruments and kits were performed according to the manufacturer’s instructions and protocols. The PCR mixture (10.0 µL 2×SYBR Green Realtime Master Mix, 0.8 µL qPCR forward primer, 0.8 µL qPCR reverse primer, 6.4 µL ultrapure H2O, and 2.0 µL Template) was added to the qPCR reaction plate. The cycling parameters were as follows: 95 °C for 30 s; followed by 30 cycles of 95 °C for 3 s and 60 °C for 30 s. The qPCR experiment was repeated more than three times. After the reaction, we exported the data from the ABI7500 Real-Time PCR machine and analyzed the data using the 2^-ΔΔCT^ method.

### 2.4 Subcellular localization analysis

*N. benthamiana* leaf epidermal cells were used for subcellular localization of MaABI5 and MaABF1. The open reading frames (ORFs) of *MaABI5* and *MaABF1* were amplified using polymerase chain reaction (PCR). Subsequently, the PCR products were introduced into the YFP-2300 vector at the XbaI and BamHI restriction sites. This process resulted in the creation of two constructs: 35Spro : MaABI5-YFP and 35Spro : MaABF1-YFP, respectively. Recombinant vectors were introduced into Agrobacterium (*Agrobacterium tumefaciens*) strain GV3101. The Agrobacterium culture was incubated at 28°C overnight, and the cells were collected by centrifugation at room temperature for 8 minutes at 2000g. The pellet was then resuspended in a buffer containing 10 mM MgCl2, 10 mM MES, 200μM acetosyringone, at pH 6.3 to achieve a final OD600 of 0.8. Agrobacterium cell suspensions were infiltrated into tobacco leaves. After 48h, laser confocal fluorescence microscopy (Olympus, Tokyo, Japan) was used to observe fluorescence signal in tobacco epidermal cells.

### 2.5 Virus-induced gene silencing

PCR amplification was performed to obtain fragments specific to *NbPDS*, *NbABF1*, and *NbABI5*, with lengths of 500 bp, 404 bp, and 329 bp, respectively. These fragments were individually inserted into the pTRV2 vector. Subsequently, the modified pTRV2 vectors were introduced into the Agrobacterium strain GV3101. The primers used in this study are listed in **Supplementary Table 1**. The relevant Agrobacterium colonies were then cultured overnight in LB medium containing 50 mg/mL kanamycin, and 50 mg/mL rifampicin, and 50 mg/mL streptomycin Sulfate. Once the concentration of Agrobacterium reached OD of 0.6 at 600 nm, we harvested the cells through centrifugation at 2000 g for 8 min. The resulting pellet was then re-suspended in a buffer containing 10 mM MgCl_2_, 10 mM MES, 200 μM acetosyringone, at a pH of 6.3. pTRV1 and pTRV2 cultures were mixed in a 1:1 (v/v) ratio and then mixture was placed in the dark for 3h at room temperature.

### 2.6 Electrophoretic mobility shift assays

The functional structural domain sequences of MaABF1 and MaABI5 were amplified by PCR and named as BRLZF1 and BRLZI5, respectively. BRLZF1 and BRLZI5 were amplified into the pET28a vector and expressed in *E. coli* BL21 (DE3) cells. Recombinant proteins were purified and used for EMSAs along with biotin-labeled fragments (~50 bp) of the *MaJOINTLESS* promoters. The same fragment, but unlabeled, was used as a competitor, while a probe within the mutant ABRE-binding cis-elements (interchanging G with C or C with G) was used as a mutant competitor in the assay. The binding of protein and nucleic acid was verified with chemiluminescence kit (Beyotime, Shanghai, China).

### 2.7 Dual luciferase reporter assays

Diagrams of the effector and reporter vectors used for dual-luciferase reporter assays are shown in **Supplementary Figure 4**. The effector and reporter plasmid constructs were co-transformed into tobacco leaves using Agrobacterium infiltration. After 2–3 d, activities of the LUC and REN luciferases were measured using the Dual Luciferase Reporter Gene Assay Kit (YEASEN, Shanghai, China). The results were calculated as the ratio of fluorescence of LUC to that of REN. Three leaves from each tobacco plant were injected. For each technical replicate, three tobacco plants were used and three technical replicates were performed.

### 2.8 Transcriptome sequencing and analysis

The samples were sequenced and analyzed by Beijing Genomics Institution (BGI, Shenzhen, China), using the BGISEQ-500 platform. The raw data obtained from sequencing were filtered to remove low quality, junctional contamination, and reads with too high an unknown base N content. The clean reads were then compared to the reference genome for subsequent analysis.

### 2.9 DEGs expression profiling and MapMan analysis

The DEseq2 method was used to detect DEGs between samples (Love et al., 2014). The DEGs were functionally classified according to Gene Ontology (GO) and Kyoto Encyclopedia of Genes and Genomes (KEGG) annotation results and official classification, while enrichment analysis was performed using the phyper function in R software. The P-value was then FDR-corrected, and functions with Q-value ≤ 0.05 were considered significantly enriched.

To determine the metabolic pathways and regulatory processes associated with the white mulberry gene, MapMan tool was downloaded from https://mapman.gabipd.org/. Since it did not provide the map files of mulberry, the proteome files were uploaded to the MapMan genome annotation tool, Mercator v3.6 https://www.plabipd.de/portal/mercator-sequence-annotation.This step allowed us to obtain the mapping result file, which was then uploaded to the MapMan local software. The mapping process utilized the log_2_FC values of DEGs (Thimm et al., 2004).

The raw sequencing data were submitted to the NCBI sequence read archive (SRA) database with the accession number: PRJNA818862.

### 2.10 Data analysis

Data analysis and mapping were performed with the GraphPad Prism 9.5.1. All data are expressed as the mean ± standard deviation. An unpaired two-tailed Student’s t-test was used to determine statistical significance. p < 0.05 was considered to indicate a significant difference (*p < 0.05, **p < 0.01, and ***p < 0.001).

## 3 Results

### 3.1 Activation of *MaJOINTLESS* promoter activity by ABA

To examine the effects of different plant hormones and abiotic stresses on *pMaJOINTLESS* activity, tobacco plants were treated with light, darkness, water, ABA, GA3, ethephon, and 2,4-D. With GUS chemical staining leaves were not stained blue after injection of clear water, indicating that clear water had no GUS activity (**Figure 1B**). However, after injection of the positive control agrobacterium bacterium solution, they were stained blue under all seven treatments, indicating that the 35S promoter had GUS activity (**Figure 1C**). The leaves of the experimental group were stained with different shades of blue after injection of the agrobacterium bacterium solution, indicating that the *pMaJOINTLESS* promoter also had GUS activity and the promoter activity intensity varied for different stress treatments (**Figure 1D**). Compared with the light blue color presented by the water treatment, the four plant hormones showed a darker blue color after treatment, with the leaves stained the most deeply under ABA treatment, followed by ethephon and GA3, and 2,4-D with a lighter color.

### 3.2 Expression patterns of *MaABF1* and *MaABI5* genes

**Figure 2:**
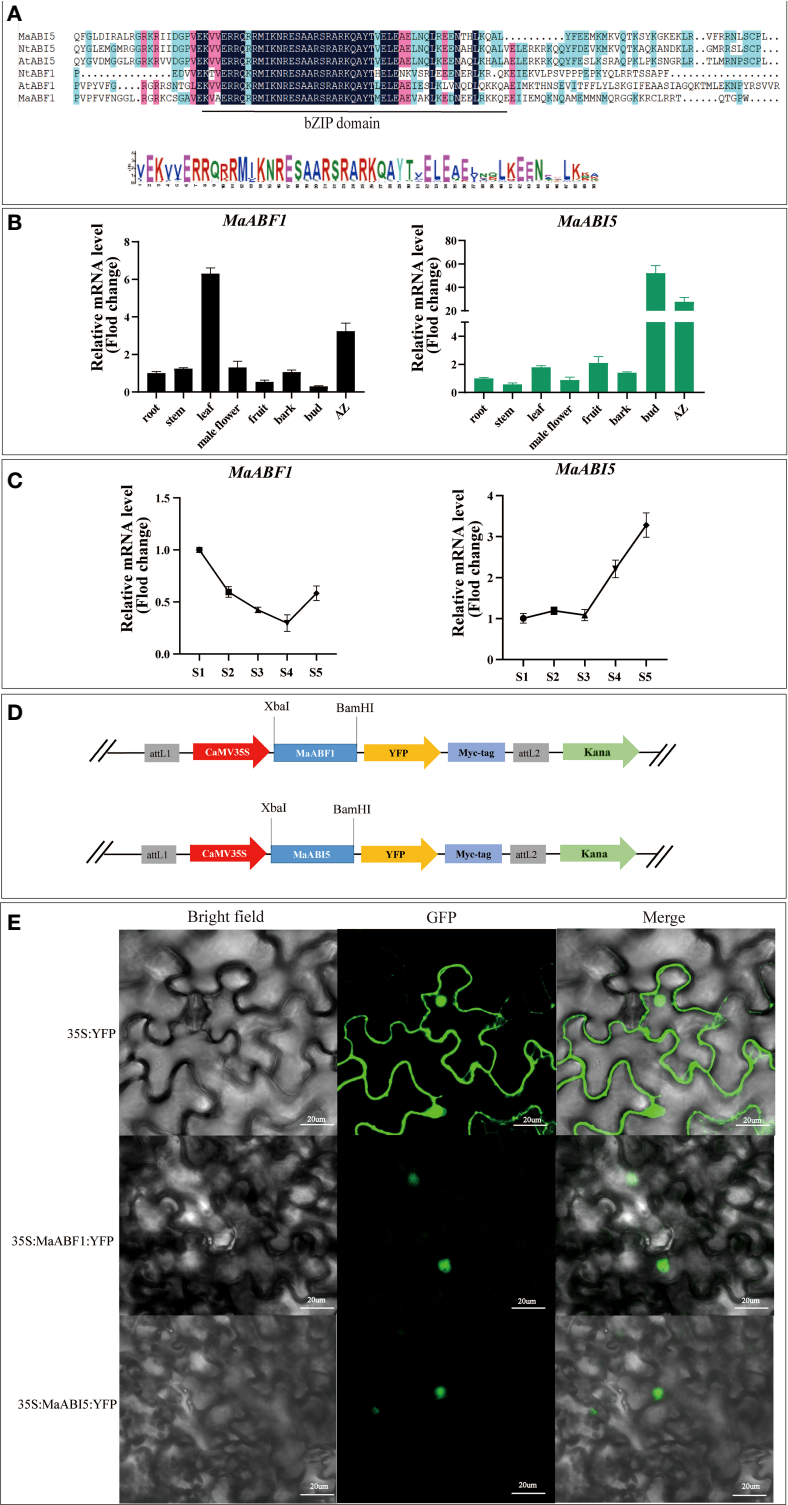
Expression patterns of *MaABF1* and *MaABI5* genes and analysis of subcellular localization of MaABF1 and MaABI5 proteins. **(A)** Sequence alignment analysis of bZIP structural domains in different species: Arabidopsis (*AtABF1* and *AtABI5*), Tobacco (*NtABF1* and *NtABI5*). **(B)** Quantitative analysis of the relative expression of *MaABF1* and *MaABI5* gene in different tissues. **(C)** Quantitative analysis of the relative expression of *MaABF1* and *MaABI5* gene in different periods. S1: Green Period. S2: Green to red period. S3: Red Period. S4: Red to purple period. S5: Purple ripening stage. **(D)** Schematic diagram of the construction of subcellular localization vectors for MaABF1 and MaABI5 proteins. **(E)** Subcellular localization of nuclear localization of MaABF1-YFP and MaABI5-YFP in tobacco leaves. Scale bars are 20 μm.

The promoter analysis of the *MaJOINTLESS* gene, suggested presence of many transcription factors including *ABF1* and *ABI5*. *ABF1* and *ABI5* are downstream transcription factors of the ABA signaling pathway (Fujita et al., 2011). Since *MaJOINTLESS* promoter activity is sensitive to ABA. Due to this ABF1 and ABI5 were selected for detailed study. The multiple sequence alignment of ABF1 and ABI5 protein sequences of *Arabidopsis* and tobacco showed that both have bZIP structural domains, thus indicating that these belong to the bZIP family of proteins (**Figure 2A**).

According to qPCR analysis, *MaABF1* and *MaABI5* genes were expressed in all tissues, *MaABF1* was higher in the AZ and leaves, and *MaABI5* was higher in the AZ and winter buds (**Figure 2B**). Temporal expression analysis showed that the expression of *MaABF1* in the AZ exhibited a decline starting from the S1 stage. The expression reached its lowest level during the S4 stage, followed by an increase in expression, While the expression of *MaABI5* in the fruit stalk displayed a gradual change from the S1 to S3 stage, and subsequently exhibited a significant increase after the S3 stage, continuing until full maturity (**Figure 2C**). These results suggest that both *MaABF1* and *MaABI5* genes play important roles in mulberry fruit abscission as they are highly expressed in the abscission zone. The opposite expression trends of these two genes during mulberry fruit ripening tentatively suggest that they may have opposite roles in the process. The subcellular localization of MaABF1 and MaABI5 proteins in tobacco tissues was examined by constructing plant subcellular localization vectors (**Figure 2D**). The results showed that green fluorescence was present throughout the tobacco cells in the control group, whereas in the experimental group, the fluorescent signal was only observed in the nucleus (**Figure 2E**). These findings suggest that MaABF1 and MaABI5 proteins are localized in the nucleus.

### 3.3 MaABF1 and MaABI5 regulate *MaJOINTLESS*

**Figure 3:**
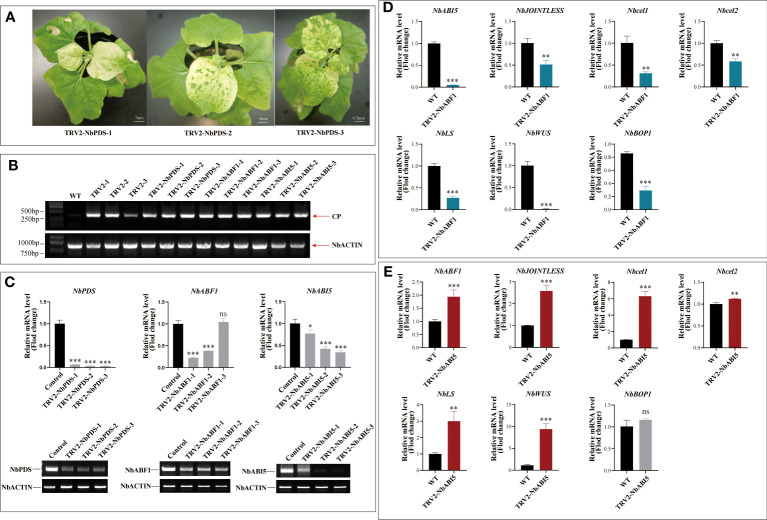
Effect of silencing the endogenous gene in tobacco on genes related to fruit abscission. **(A)** Phenotypic observations after 15 days of Virus-induced gene silencing of the *PDS* gene in *N. benthamiana.* **(B)** Semi-quantitative detection of viral capsid protein CP protein expression in each sample. **(C)** Real-time fluorescence quantification and semi-quantitative detection of the expression of silenced genes in each silenced Plants. **(D)** Detection of expression of abscission -related genes on *NbABF1*-Silenced Plants. **(E)** Detection of expression of abscission-related genes on *NbABI5*-Silenced Plants. Data are means ( ± SE) from three replicates. Significant differences between means were determined using Student’s *t*-test: *P<0.05, **P<0.01. ***p < 0.001, ns means no significant difference.

To verify whether MaABF1 and MaABI5 can bind to the *MaJOINTLESS* promoter, structural domain proteins BRLZF1 and BRLZI5 were expressed in a prokaryotic expression system (**Supplementary Figure 1**). The proteins were incubated with probes for EMSA experiments, and binding bands were observed in both experimental groups compared to the negative control (no target protein), indicating that the structural domain proteins BRLZF1 and BRLZI5 can bind to the *MaJOINTLESS* promoter (**Figure 3A**). Since BRLZF1 and BRLZI5 are tagged with Flag, we used the Flag antibody to bind competitively to the proteins, forming a supershift band. As the antibody concentration increased, the antibody-protein binding band strengthened while the probe-protein binding band weakened (**Figure 3B**). Next, we synthesized unlabeled biotin probes as cold probes, diluted them to 5×, 10×, and 25×, and incubated them with the proteins. We found that the binding of the biotin-labeled probe to the protein weakened as the cold probe concentration increased (**Figure 3C**). Finally, mutant probes were synthesized with biotin markers and incubated them with the proteins. No band was detected in the mutant group, indicating that MaABF1 and MaABI5 are specific to the predicted binding site of the *MaJOINTLESS* promoter (**Figure 3D**).

**Figure 4:**
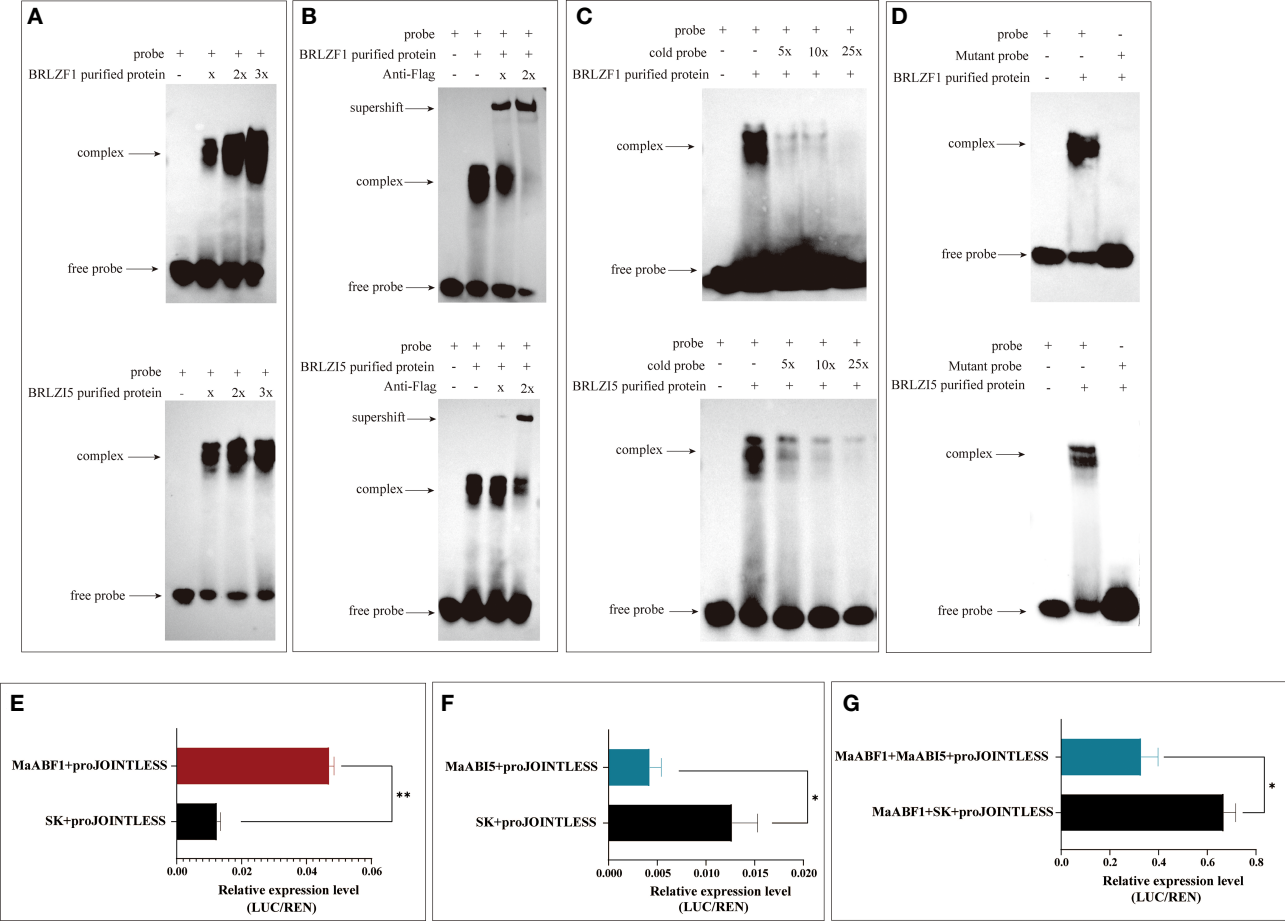
The association of MaABF1and MaABI5 with the *MaJOINTLESS* promoter *in vitro*. **(A)** EMSA binding assay with biotin probes to target proteins. **(B)** Antibodies are used to compete for the binding of biotin probe to the protein. **(C)** Use non-biotin-labeled probe (cold probe) to compete for the binding of biotin probes to proteins. **(D)** EMSA binding assay with mutant probes to target proteins. Shifted bands, suggesting the formation of DNA–protein complexes, are indicated by arrows. ‘–’ and ‘+’ represent an absence or presence, respectively. ‘x’ ‘2x’indicates increasing amounts of unlabeled or mutated probes introduced for competition. **(E)** *MaABF1* activates the expression of *MaJOINTLESS*) *in vivo* as indicated by transient dual-luciferase reporter assays in tobacco leaves, indicated by the ratio of LUC to REN expression. **(F)** *MaABI5* suppresses the expression of *MaJOINTLESS in vivo.* **(G)** Co-expression of MaABF1 and *MaABI5* suppresses *MaJOINTLESS* promoter expression *in vivo*. Data are means ( ± SE) from three replicates. Significant differences between means were determined using Student’s *t*-test: *P<0.05, **P<0.01.

To verify the regulatory role of MaABF1 and MaABI5 on the *MaJOINTLESS* promoter, we performed a dual-luciferase assay. *MaABF1* and *MaABI5* were separately constructed onto the pGreenII62-SK vector, while the *MaJOINTLESS* promoter was ligated to the pGreenII0800-LUC vector, and then transformed into Agrobacterium. After expression in injected tobacco leaves, the enzyme activity ratios of firefly luciferase and sea kidney luciferase were measured. The results showed that *MaJOINTLESS* activity was significantly enhanced when MaABF1 was expressed, indicating that MaABF1 could transcriptionally activate *MaJOINTLESS* (**Figure 4E**). On the other hand, when MaABI5 was expressed, *MaJOINTLESS* activity was significantly reduced, indicating that MaABI5 could transcriptionally repress *MaJOINTLESS* (**Figure 4F**). Interestingly, when both MaABI5 and MaABF1 were present, *MaJOINTLESS* activity was significantly reduced, suggesting a transcriptional repression effect (**Figure 4G**). We speculate that MaABI5 and MaABF1 may form a transcriptional complex, preventing MaABF1 from binding to the promoter region and leading to loss of its activation effect.

Further, *MaABF1* and *MaABI5* were silenced in tobacco using VIGS. Following 15 days silencing of *NbPDS* gene in tobacco, the newly grown leaves of all three positive plants showed varying degrees of albinism, indicating that the positive plants were already prone to gene silencing (**Figure 3A**). The new leaves of each silenced strain were collected, and wild-type tobacco was used as a control. It was found that all silenced strains expressed the CP protein of the viral capsid, while the wild type did not show the CP protein. *ACTIN* (GenBank: AY594294.1) was used as an internal reference gene. This indicates that the virus was successfully expressed in tobacco leaves (**Figure 3B**). To assess the effect of gene silencing, qPCR was used to determine the relative expression of *NbPDS*, *NbABF1*, and *NbABI5*. The results are shown in **Figure 3C**. *NbPDS* was significantly down-regulated in all positive silencing strains; *NbABF1* was significantly down-regulated in TRV2-NbABF1-1 and TRV2-NbABF1-2, while no significant difference was found in TRV2-NbABF1-3; TRV2-NbABI5-2 and TRV2-NbABI5-3 had stronger silencing effects. qRT-PCR results were consistent with the semi-quantitative-PCR results.

To confirm the effect of *NbABF1* on fruit abscission, the expressions of *NbABI5*, *NbCel1*, *NbCel2*, *NbJOINTLESS*, *NbLs*, *NbWUS*, and *NbBOP1* were examined in the TRV2-NbABF1-1 strain. The results showed that the downregulation of *NbABF1* led to a decrease in the expression of *NbABI5*, *NbJOINTLESS*, *NbCel1*, *NbCel2*, *NbLs*, *NbWUS*, and *NbBOP1* (**Figure 3D**). This indicates that *NbABF1* promotes the expression of fruit abscission-related genes. To verify the effect of *NbABI5* on fruit abscission, we measured the expression of abscission-related genes in wild-type and silenced strains. The results are shown in **Figure 3E**. The downregulation of the *NbABI5* gene led to an up-regulation of *NbABF1*, *NbJOINTLESS*, *NbCel1*, *NbCel2*, *NbLs*, *NbWUS*, and *NbBOP1* expression suggestings that NbABI5 inhibits the expression of fruit abscission-related genes.

**Figure 5:**
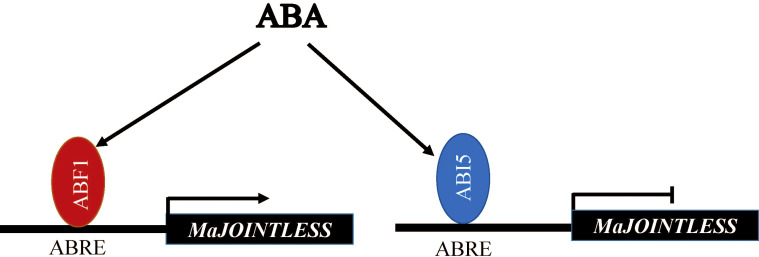
Hypothetical model showing the role of MaABI5 and MaABF1 in the regulation of *MaJOINTLESS*.

Our results demonstrate that MaABF1 and MaABI5 regulate the expression of *MaJOINTLESS*, positively and negatively, respectively (**Figure 5**).

### 3.4 Transcriptome analysis of mature-fruit abscission in mulberry

**Figure 6:**
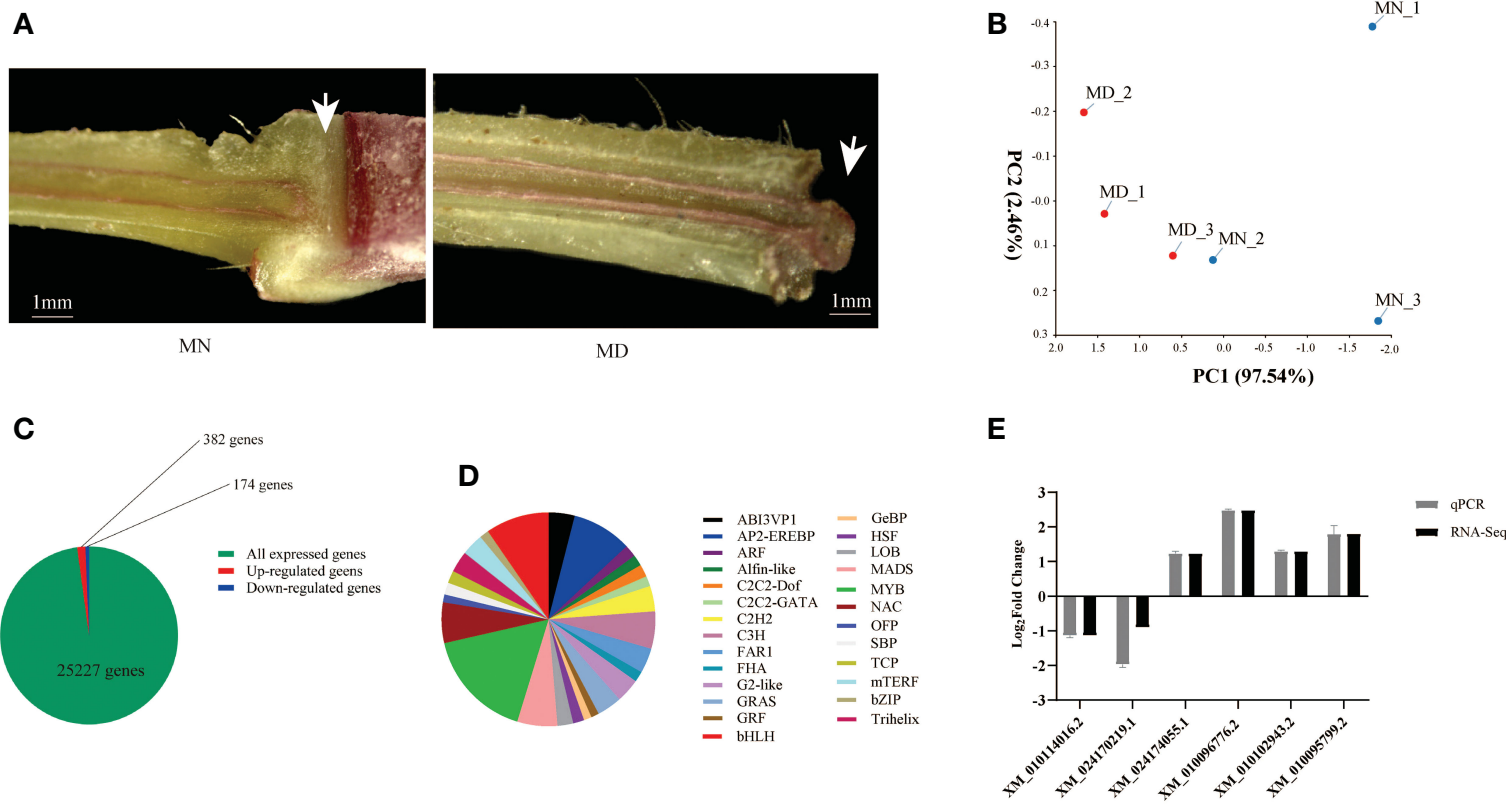
Transcriptome sequencing analysis of MD and MN. **(A)** Longitudinal sections of fruit stalk stained with phloroglucinol, showing the presence of the AZ in MN and MD. Scale bars are1mm. **(B)** Principal component analysis of Transcriptomic data of MD and MN. **(C)** Number of differentially expressed genes in MFA of mulberry. DEGs were screened using |log2foldchange| ≥1 and P-value ≤ 0.01 as thresholds. **(D)** Transcription factor analysis of MFA. **(E)** qPCR Validation of Transcriptome Sequencing Data. The white arrow in the left panel of **(A)** represents the nonlignified portion, and the structure is shed at maturity.

It has been shown that *J* mutants inhibit the development of AZ at the stem of tomato, exhibiting significant phenotypic differences from wild-type AZ (Roldan et al., 2017). Our results found that the longitudinal section of normal fruit stalks stained with phloroglucinol showed non-lignified structures compared to deciduous fruit stalks, and this structure is the AZ organization (**Figure 6A**). Transcriptome sequencing of mulberry AZ was performed to obtain genome-wide gene expression profiles of mulberry AZ tissues at the mature stages.

PCA was conducted to confirm the differences between the MN and MD samples, and the variance explained by the first two principal components was determined (97.54% and 2.46%, respectively). The results showed that there were differences in the MN and MD sample groups, and each sample formed a separate cluster (**Figure 6B**).

A total of 25,227 genes were identified in the stalk tissue of young mulberry fruits. To compare gene expression differences between fallen and normal fruit, DEGs were screened between fallen and normal fruit stalks at maturity using |log2foldchange| ≥ 1 and P-value ≤ 0.01 as the threshold. Among them, 556 DEGs were identified after fruit drop at maturity, of which 382 genes were up-regulated and 174 genes were down-regulated. Of all expressed genes, only 2.2% were differentially expressed (**Figure 6C**). Analysis of TFs showed that 26 transcription factors are involved in the process of mulberry ripening fruit abscission, with MYB, bHLH, AP2-EREBP, and MADS accounting for the largest proportion (**Figure 6D**). We found that the transcription factors involved in mature fruit abscission in mulberry were like those of other model plants.

To verify the authenticity of RNA-seq gene expression data obtained qRT-PCR analysis of six randomly selected DEGs was performed. *PRR37* (XM_010114016.2) and alpha-galactosidase 1 (XM_024170219.1) were down-regulated, while glucan endo-1,3-beta-glucosidase (XM_010102943.2), *EP3* (XM_010095799.2), germin-like protein subfamily 1 member 17 (XM_024174055.1), and *HSPRO2* (XM_010096776.2) showed up regulation. These results were consistent with the RNA-Seq data (**Figure 6E**).

**Figure 7:**
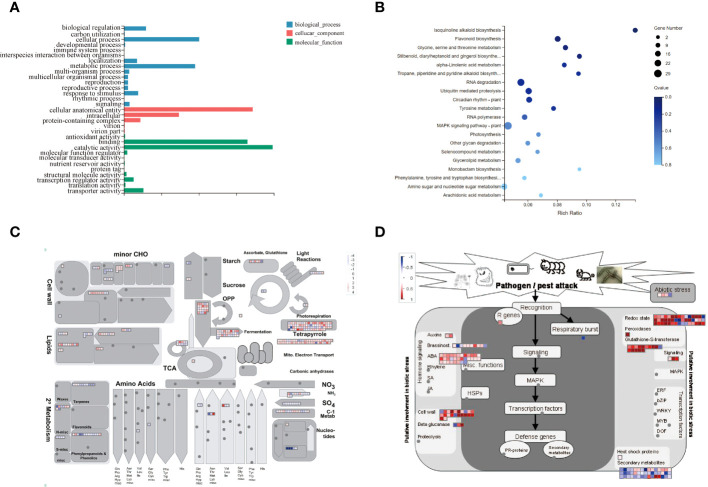
DEGs Expression Analysis. **(A)** GO Enrichment Analysis of Differentially Expressed Gene Functions. **(B)** Enrichment Analysis of the KEGG Metabolic Pathway of DEGs. **(C)** MapMan analysis of metabolic pathways in mulberry. **(D)** MapMan analysis of differential gene expression in white mulberry in response to biotic stress. Log_2_FC values of differentially expressed genes of MFA were substituted into MapMan native software to visualize the response to biotic stress map. Darker red indicates more significant up-regulation of expression compared to MD, darker blue indicates more significant down-regulation of expression compared to MD.

The GO classification of DEGs in mulberry fruit abscission can be categorized into three categories: biological processes, cellular components, and molecular functions (Gaudet et al., 2021). Significantly enriched genes include those involved in cellular and metabolic processes, cellular anatomical entities and intracellular genes, catalytic activity, and binding genes. The most abundant genes were those involved in catalytic activity, suggesting that many important enzymes may be involved in the fruiting process of mulberry, resulting in a series of catalytic reactions (**Figure 7A**).

The KEGG pathway enrichment analysis showed that most of the DEGs were concentrated in the MAPK signaling pathway, flavonoid biosynthesis, RNA degradation, circadian rhythm-plant, glycine, serine and threonine metabolism, isoquinoline alkaloid biosynthesis, and other pathways. Additionally, the amino sugar and nucleotide sugar metabolism were also involved in the abscission of mulberry fruit (**Figure 7B**). Importantly, all genes involved in this pathway were upregulated (**Supplementary Table 2**; **Table 3**). Therefore, we speculated that the MAPK signaling pathway and flavonoid biosynthesis pathway may affect the shedding of mature mulberry fruits.

The MapMan pathway annotator was used to outline the metabolism of DEGs (**Figure 7C**). DEGs were mainly expressed in secondary metabolism, minor CHO, lipid, fermentation, TCA, and light response pathways. Interestingly, most DEGs were up-regulated during metabolism, while a few genes were down-regulated in expression during MFA of mulberry. All DEGs in the transcriptome were transformed into MapMan-recognized IDs, and the expression of each DEG in response to biotic stress was expressed by heatmap representation. It can be found that most of the up-regulated DEGs are concentrated in auxins, ABA, cell wall, beta glucanase, glutathione-S-transferase, redox state, and signaling genes, while a few down-regulated genes encode secondary metabolites and respiratory burst gene (**Figure 7D**).

These results predict that DEGs have critical roles in fruit abscission mechanism. Seven DEGs having high expression in fruit abscission (germin-like proteins (GLPs), pathogen infection-related *NHL* (*NDR1*/*HIN1*-like) family genes, the amphiphysin-like protein 1 (ALP1), glucosidase genes, copine family gene *BON1*, and acidic endogenous chitinase-like genes) were identfied. These results provide a reference for subsequent in-depth studies on the molecular mechanism of fruit abscission in mulberry.

## 4 Discussion

The shedding of organs is necessary for normal plant development and survival, Fruit abscission in plants is the result of a combination of environmental and physiological factors. Here, we report the mechanism of *ABF1/ABI5* and *MaJOINTLESS* regulation during fruit abscission in mulberry. The expression of abscission-related genes was measured in the AZ of the fruit stalk during the ripening and abscission of mulberry by transcriptome sequencing.

The exogenous application of ABA is well-known to promote organ abscission or senescence in plants. For example, spraying ABA in tomatoes increased the content of ABA and ETH synthesis in the fruit and accelerated the ripening of tomatoes (Berry and Bewley, 1991). In addition, exogenous application of ABA in strawberry promoted fruit softening and color change (Jia et al., 2011) and in grapes induced the expression of proteins related to grape ripening (Giribaldi et al., 2010). Further, same results have been reported in banana for exogenous application of ABA (Jiang et al., 2000). ABA has diverse roles in biotic and abiotic stress tolerance in plants. For example, after water stress, the ABA content of maize leaves increased (Wang et al., 2008). In addition, ABA is involved in the regulation of heat tolerance in plants and played a crucial roles in adaptive responses to drought, high salt, and freezing to induce cellular osmotic stress. These changes which in turn stimulated ABA accumulation in nutrient tissues (Zhu, 2002; Larkindale et al., 2005; Seki et al., 2007; Rezaul et al., 2019; Zhang et al., 2019).

A detailed promoter analysis of *MaJOINTLESS* gene was performed to investigate the upstream regulatory factors. Results suggested that the *MaJOINTLESS* promoter exhibits ABA-responsive activity (**Figure 1**) and two interesting transcription factors, ABF1/ABI5 were identified. These two regulatory factors are downstream transcription factors in the ABA pathway. Multiple sequence alignment analysis revealed that both MaABF1 and MaABI5 contained the BRLZ domain (**Figure 2A**), suggesting that these are members of bZIP transcription factor family (Dröge-Laser et al., 2018).

It has been reported that bZIP is involved in the ABA signaling pathway, such as the apple *MdbZIP*44 gene that promotes anthocyanin accumulation in response to ABA (An et al., 2018). bZIP binds to the promoter of the *PP2C* gene in response to the ABA signaling pathway (Wang et al., 2019). In addition, bZIP is involved in abiotic stresses (Fujita et al., 2013). The bZIP gene *ABF1* in *Arabidopsis* is required for seedling growth in winter and has a regulatory role for seed dormancy and germination (Sharma et al., 2011). The potato bZIP gene *ABI5* can respond to ABA or osmotic stress (Liu et al., 2019); the barley bZIP gene *HvABI5* has the ability to regulate drought response and seed germination (Collin et al., 2020); bZIP can also respond to ABA signals by regulating the plant biological clock (Yang et al., 2021). MaABF1 and MaABI5 were localized in the nucleus (**Figure 2E**). Our results are consistent with the previous findings that the bZIP transcription factors are localized in the nucleus (Liang et al., 2022; Liu et al., 2022; Wang et al., 2022).

We found that the promoter region of the *MaJOINTLESS* gene contains elements associated with plant hormones, such as the abscisic acid response element ABRE (**Supplementary Figure 2**). The bZIP family transcription factors have been found to bind to the ABA response element (ABRE) (Choi et al., 2000). Therefore, we hypothesized that the transcription factors MaABF1 and MaABI5 could bind to the *MaJOINTLESS* promoter on the ABRE element. EMSA experiments were performed, which revealed that MaABF1 and MaABI5 could bind specifically to the predicted binding sites on the *MaJOINTLESS* promoter (**Figures 4A–D**). ABI5 is a key transcription factor that regulates the expression of ABA-responsive genes in the ABA signaling pathway (Skubacz et al., 2016), while ABF1 is an ABA-binding factor/ABA-responsive element binding protein that plays a role in *Arabidopsis* seed germination, plant growth, and development (Fernando et al., 2018).

Our findings showed that MaABI5 acted as a negative regulator, repressing the expression of *MaJOINTLESS* and other abscission-related genes. On the other hand, *MaABF1* acted as a positive regulator, promoting the expression of *MaJOINTLESS* and other abscission-related genes. To validate this, *MaABF1* and *MaABI5* were overexpressed in tobacco and found that *MaABF1* positively regulated *NbJOINTLESS*, whereas *MaABI5* negatively regulated it (**Supplementary Figure 3**). Notably, when the homologous gene *NbABF1* was silenced in tobacco, *NbABI5* and *NbJOINTLESS* showed downregulation. Conversely, *NbJOINTLESS* showed upregulation after *NbABI5* silencing. bZIP transcription factors in *Arabidopsis* can antagonize each other for cross-regulation, such as *ABI5* repressing the expression of *ABF1* (Finkelstein et al., 2005). Therefore, we speculate that there could be a similar regulation mechanism between *NbABF1* and *NbABI5*, whereby *NbABI5* negatively regulates *NbABF1*.

Moreover transcriptome sequencing was performed to analyze the DEGs during fruit abscission in mulberry. The KEGG pathway enrichment analysis revealed that most of the DEGs were concentrated in pathways such as MAPK signaling, flavonoid biosynthesis, citric acid cycle, phytohormone signaling, and amino acid biosynthesis, indicating their significant roles in regulating fruit abscission. Metabolic pathway analysis by MapMan showed that most DEGs were up-regulated in growth hormones(ABA), cell wall, β-glucanase, glutathione-S-transferase, redox status, and signal transduction at maturity. *MaABF1*, *MaABI5*, and *MaJOINTLESS* genes were significantly up-regulated during fruit abscission at maturity, demonstrating their involvement in fruit abscission in mulberry (**Supplementary Figure 5**).

DEGs were analyzed and several regulatory factors involved in fruit abscission were detected (**Supplementary Table 4**). The GLPs family of plants are an important class of stress-responsive proteins (Membré et al., 2000; Patnaik and Khurana, 2001). Overexpression of NHL family member *NHL6* regulated seed germination under abiotic stress by affecting ABA biosynthesis and signaling (Bao et al., 2016). The disease-course-related gene *HIN1* may enhance plant resistance by activating the jasmonic acid signaling pathway (Peng et al., 2019). β-glucosidases (BGs) are the main regulatory enzymes of abscisic acid reactivation (Lee et al., 2006). With reference to the already reported functions of these genes, we speculate that these genes might have critical roles during fruit abscission in mulberry.

## 5 Conclusions

The upstream regulators of *MaJOINTLESS* (*MaABF1* and *MaABI5*) were identified and their structural, expression, and localization analysis were performed. After this a series of experiments were performed to verify the regulatory roles of *MaABF1* and *MaABI5* on *MaJOINTLESS*. Subsequently, we demonstrated that *MaABF1* transcription activates *MaJOINTLESS*, while *MaABI5* transcription represses it, and both MaABF1 and MaABI5 proteins bind to the ABRE element upstream of the *MaJOINTLESS* promoter. Further, we analyzed the expression of differential genes during mulberry abscission at maturity by transcriptome sequencing. These DEGs are involved in pathways related to phytohormones, MAPK, flavonoids, citric acid, hydrolases, transporter proteins, and other substances synthesis. We also screened some factors that may be related to fruit drops in mulberry to provide a reference for subsequent in-depth studies and next experiments on physiological fruit drop in mulberry.

## Conflicts of interest

The authors declare that the research was conducted in the absence of any commercial or financial relationships that could be construed as a potential conflict of interest.

## Data availability statement

The data presented in the study are deposited in the NCBI sequence read archive (SRA) database repository, accession number PRJNA818862.

## Author contributions

XD conceived and wrote the original manuscript. XD and JD worked on the experimental part under the guidance of XZ and YP. BA revised the English grammar of the manuscript. ZF and XL critically reviewed the draft. LL performed the statistical analysis of the experimental data. All authors contributed to the article and approved the submitted version.
