# Supplementary material for: MaABI5 and MaABF1 transcription factors regulate the expression of MaJOINTLESS during fruit abscission in mulberry (Morus alba L.)
Source: Front Plant Sci. 2023 Aug 21;14:1229811. doi: 10.3389/fpls.2023.1229811 (PMC10475957; doi:10.3389/fpls.2023.1229811)
Supplement: Supplementary file 1 [file DataSheet_1.docx]

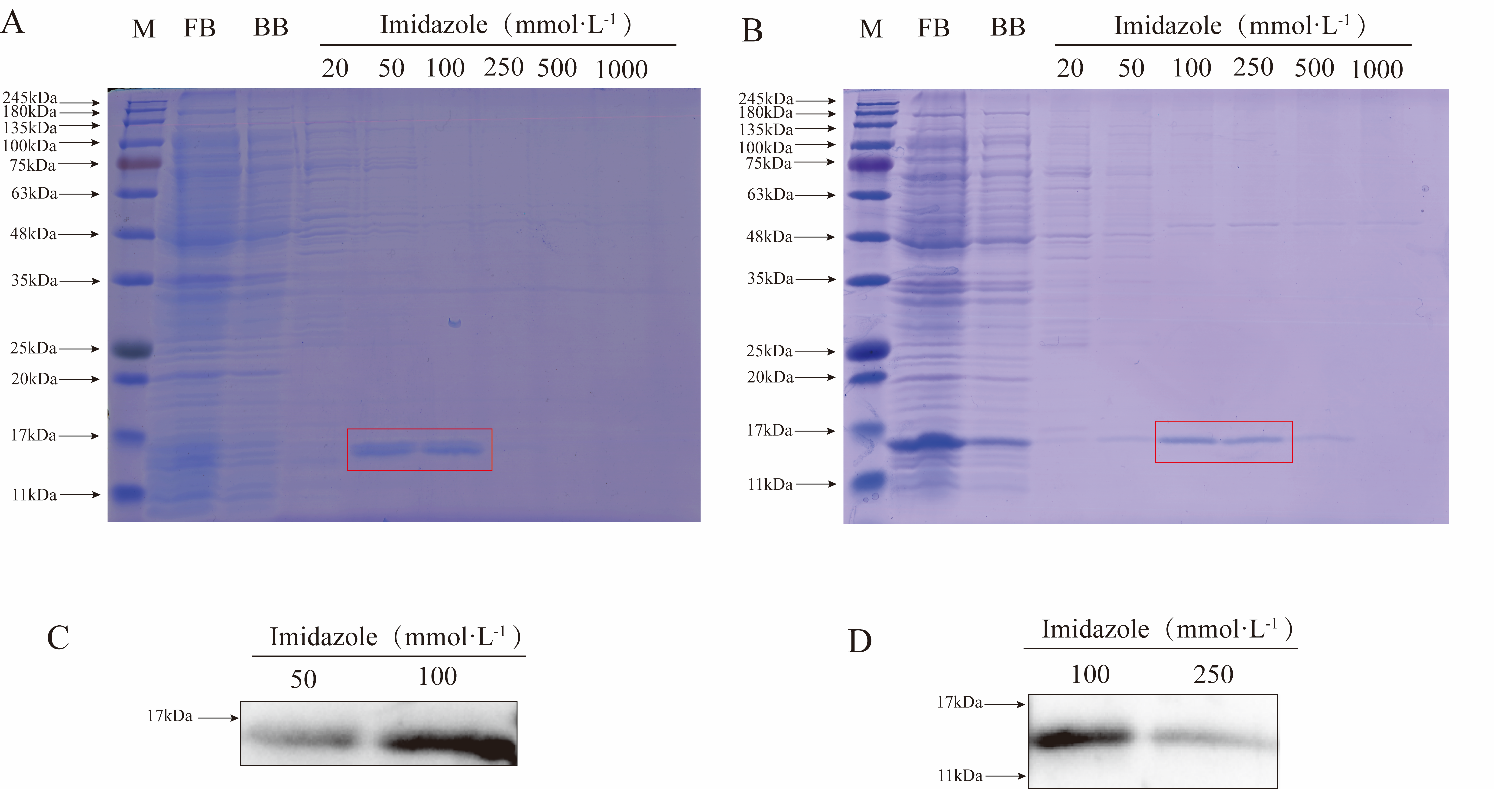


**Supplementary Figure 1**. Prokaryotic expression and analyses of the structural domain proteins BRLZF1and BRLZI5. (A-B): SDS-PAGE analysis of BRLZF1(A) and BRLZI5(B); (C-D): Western blot analysis of purified protein BRLZF1(C) and BRLZI5(D); M: molecular size markers; FB: flow-through buffer; BB: binding buffer.


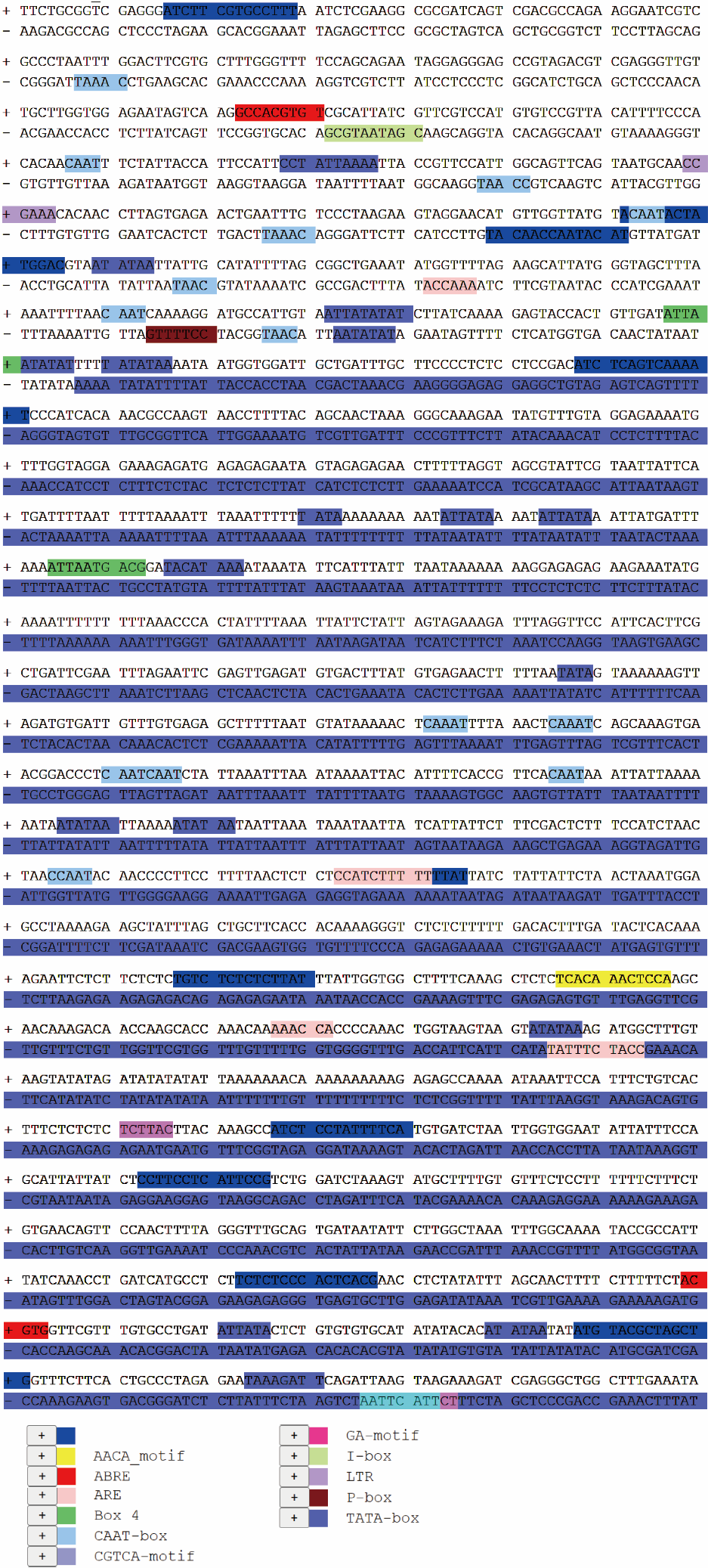


**Supplementary Figure 2**. Analysis of the cis-acting element of the MaJOINTLESS promoter. The red part represents ABRE components.


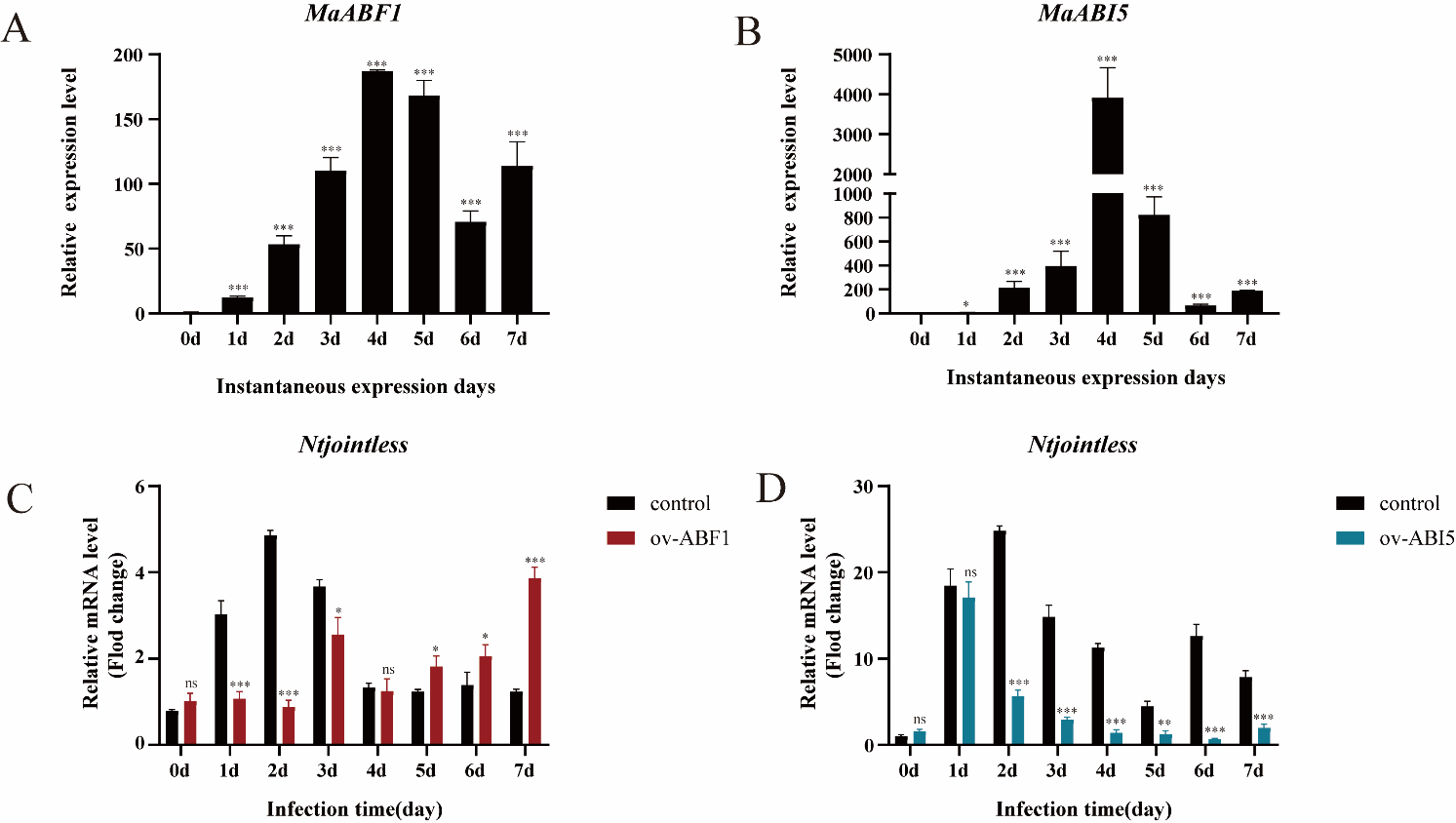


**Supplementary Figure 3.** Expression pattern analysis of *MaABF1* and *MaABI5* after transient overexpression. A-B: The expression of *MaABF1* and *MaABI5* genes in tobacco within one week; C-D: The expression of *NbJOINTLESS* within one week after transient expression of *MaABF1* and *MaABI5* genes. Three groups were repeated to take the mean, and the control group mean was used as the normalization factor for normalization, T-test, ns means no significant difference, * indicates significance level p<0.05, ** indicates significance level p<0.01, *** indicates significance level p<0.001.


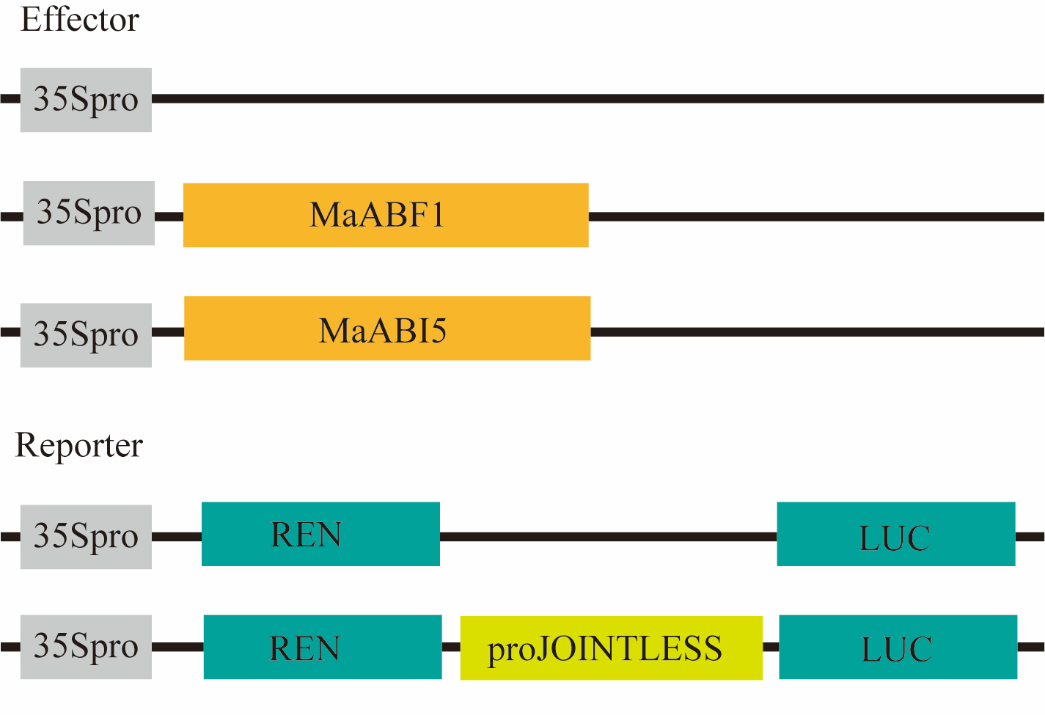


**Supplementary Figure 4.** Construction of double luciferase vector.


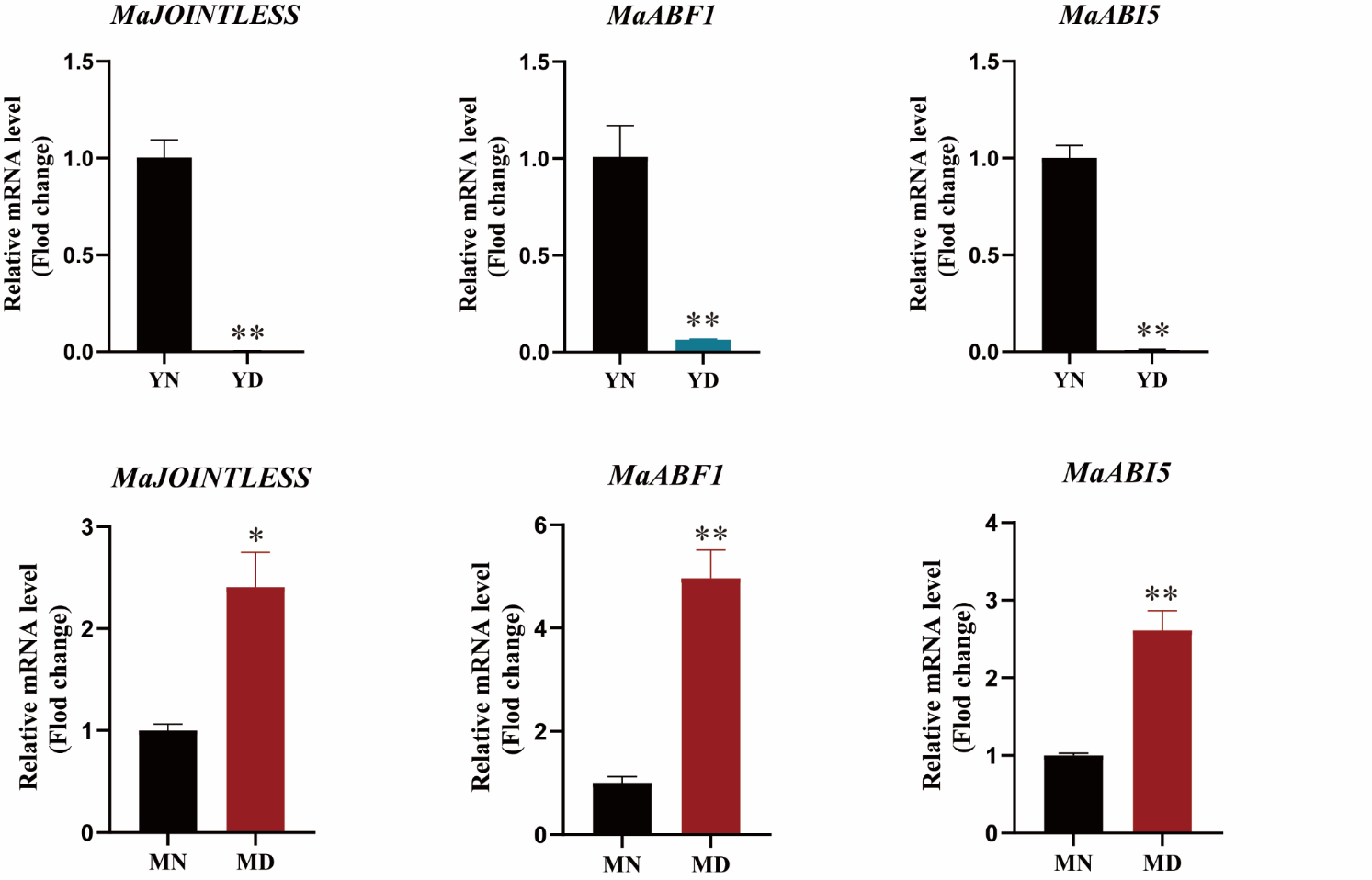


**Supplementary Figure 5.** Relative expression of *MaABF1*, *MaABI5* and *MaJOINTLESS* genes.

**Supplementary table 1.** Primers used in this study.

| Primer name | Sequence（5'-3'） | Purpose | |
| --- | --- | --- | --- |
| MaJOINTLESS-F | ATGGCGAGAGAGAAGATTCAG | Gene clone | |
| MaJOINTLESS-R | TCAGCCAGAGTATGGTAGCC |  |  |
| pMaJOINTLESS-F | TGGTTTTAGAAGCATTATGGGTAG |  |  |
| pMaJOINTLESS-R | TAATCTGAATCTTCTCTCTCGCCAT |  |  |
| MaABF1-F | ATGGGGTCAAATATGAACTTTAAGA |  |  |
| MaABF1-R | TTACCAAGGGCCAGTGAGCGTCCT |  |  |
| MaABI5-F | ATGGTGGTTCCGGAGCCGGAAATCG |  |  |
| MaABI5-R | TCACAAAGGGCAGCTCAAATTCCTT |  |  |
| qMaActin-F | AAGTCATCACAATCGGAGC | qPCR | |
| qMaActin R | AGGGAACATAGTTGAACCA |  |  |
| qMaABF1-F | GCCGCTCTACCCCAAACCT |  |  |
| qMaABF1-R | TAACGCCGCCAGCACCTAT |  |  |
| qMaABI5-F | AACCACCCGTTCTCGTCCCTC |  |  |
| qMaABI5-R | CTCGGCGGTCCAAATGCTG |  |  |
| qMaJOINTLESS-F | TTCTCTGCGATGCCGATGTT |  |  |
| qMaJOINTLESS-R | CTTGCTCAACCTGGAGTGGT |  |  |
| 2300-YFP-F | CACAACATATACAAAACAAACGAAT | Subcellular localization analysis | |
| 2300-YFP-R | CAGGGTCAGCTTGCCGTAGGTGGCA |  |  |
| YFP-ABF1-F | **TCACCATTTACGAACGATA**TCTAGAATGGGGTCAAATATGAACTTTAAGA |  |  |
| ABF1-YFP-R | **CCCTTGCTCACCATGTTAATTAA**GGATCCCCAAGGACCTGTGAGCGTCCT |  |  |
| YFP-ABI5-F | **TCACCATTTACGAACGATA**TCTAGAATGGTGGTTCCGGAGCCGGAAATCG |  |  |
| ABI5-YFP-R | **CCCTTGCTCACCATGTTAATTAA**GGATCCCAAAGGGCAGCTCAAATTCCT |  |  |
| PVCT024-pMaJOINTLESS-F | **TTGAATCTTTGACTCCATG**AAGCTTTGGTTTTAGAAGCATTATGGGTAG | Activation of *MaJOINTLESS* promoter | |
| pMaJOINTLESS- PVCT024-R | **ACTGACCACCCGGGGATCC**TCTAGATAATCTGAATCTTCTCTCTCGCCAT |  |  |
| PMa024-F | CGAGTTCTGTTAGGTCCTCTATTTG |  |  |
| PMa024-R | ACGCGCTTTCCCACCAACGCTGATC |  |  |
| BRLZF1-BamHI-flag-F | GGATCCATG*GATTACAAGGATGACGACGATAA***G**AAAGTTATTGAAAGAAGGCA | Prokaryotic expression of the structural domain proteins BRLZF1and BRLZI5. | |
| BRLZF1-NOTI-R | GCGGCCGCTTATCTATTCATTTTCTCCAAAAGC |  |  |
| BRLZI5-BamHI-flag-F | GGATCCATG*GATTACAAGGATGACGACGATAA***G**AAGGTGGTGGAGAGGCGGCAG |  |  |
| BRLZI5-NOTI-R | GCGGCCGCTCACTTCATTTCCTCAAAATACTGT |  |  |
| SK-MaABF1-F | **AGCTGAGCTCCACCGCGGTG**GCGGCCGCATGGGGTCAAATATGAACTTTAAGA | Dual Luciferase Reporter Assays | |
| MaABF1-SK-R | **GAATTCCTGCAGCCCGGG**GGATCCCTATGGAGCCGATGCCCCACAACT |  |  |
| SK-MaABI5-F | **AGCTGAGCTCCACCGCGGTG**GCGGCCGCATGGTGGTTCCGGAGCCGGAAATCG |  |  |
| MaABI5-SK-R | **GAATTCCTGCAGCCCGGG**GGATCCTCACAAAGGGCAGCTCAAATTCCTT |  |  |
| pGreen0800-LUC-F | GAATTGTAATACGACTCACT |  |  |
| pGreen0800-LUC-R | TTCTTTATGTTTTTGGCGTC |  |  |
| 62-Sk-F | TCATTTGGAGAGGACAGC |  |  |
| 62-SK-R | GTGATTTCAGCGTACCG |  |  |
| riNbPDS-F | ATGCCCCAAATCGGACTTGTATCT | Vector construction for gene silencing in tobacco | |
| riNbPDS-R | AATATGTGCAACCCAGTCTCGTACC |  |  |
| riNbABF1-F | CAACTCAAGCACAAGACCCA |  |  |
| riNbABF1-R | CACTCCAGCCTTAACCAAAA |  |  |
| riNbABI5-F | CTGCACCTCCTCAACAGCAATCA |  |  |
| riNbABI5-R | ACATACTCCATCCGACGACACCG |  |  |
| NbPDS-TRV2-F | TCTCTAGAAGGCCTCCATGGGGATCCATGCCCCAAATCGGACTTGTATCT |  |  |
| NbPDS-TRV2-R | TCTTCGGGACATGCCCGGGCCTCGAGAATATGTGCAACCCAGTCTCGTACC |  |  |
| riNbABF1-TRV2-F | TCTCTAGAAGGCCTCCATGGGGATCCCAACTCAAGCACAAGACCCA |  |  |
| riNbABF1-TRV2-R | TCTTCGGGACATGCCCGGGCCTCGAGCACTCCAGCCTTAACCAAAA |  |  |
| riNbABI5-TRV2-F | TCTCTAGAAGGCCTCCATGGGGATCCCTGCACCTCCTCAACAGCAATCA |  |  |
| riNbABI5-TRV2-R | TCTTCGGGACATGCCCGGGCCTCGAGACATACTCCATCCGACGACACCG |  |  |
| CP-F | CGGTCCTGCTGACTTGAT | Detection of Gene Silencing Plant Efficiency | |
| CP-R | TCCCTTGGTTCGTCGTAA |  |  |
| NbACTIN-F | AAAGGCTAATCGTGAAAAGA |  |  |
| NbACTIN-R | TATCGGCAATACCTGGGAAC |  |  |
| RT-NbABF1-F | GTTGCTGAGCCGACTCCAGGCAAGA |  |  |
| RT-NbABF1-R | TTAGAAAGGGGCGGAGCTTGTTCTA |  |  |
| RT-NbABI5-F | ATGGGCGTACCAGAGTCGGAGATGG |  |  |
| RT-NbABI5-R | GGGCGGCTGCATTCTCCTGTTCGCG |  |  |
| qNbActin-F | TCCTGATGGGCAAGTGATTAC |  |  |
| qNbActin-R | TTGTATGTGGTCTCGTGGATTC |  |  |
| qNbPDS-F | TTCCTCACGCCCAACTAAACC |  |  |
| qNbPDS-R | CATGCAGCTACCTTCCCACCT |  |  |
| qriNbABF1-F | ATGCCTCAGATACCACCGC |  |  |
| qriNbABF1-R | TCATCCTCTTTTGCCTCCT |  |  |
| qRiNbABI5-F | GGGTTCTCTTACACTTCCG |  |  |
| qRiNbABI5-R | GACACTGCTCCCATTATTC |  |  |
| qNbJOINTLESS-F | CCTCCTCAAGATGATGACAGT | Identification of the expression level of shedding related genes after gene silencing | |
| qNbJOINTLESS-R | TCAAGAACCATTAGCCAAACA |  |  |
| qNbcel1-F | AATCCCAATCCAAACACACA |  |  |
| qNbcel1-R | AAGCAGCCACAGATCCTACA |  |  |
| qNbcel2-F | TTTTCTTCCTCTCCGTCAATCC |  |  |
| qNbcel2-R | TAACCTCCTGTCAAGTCCACTC |  |  |
| qNbWUS-F | TTGATGAAACCCTAGTAGACGA |  |  |
| qNbWUS-R | CTGCCAATGAAAGAGTTGAGAC |  |  |
| qNbLS-F | TTCTTCTATTGTTCTTCTCCCT |  |  |
| qNbLS-R | TCATCCTCTCTCTACTGCTCGG |  |  |
| qNbBOP1-F | GAAAGGAAAGGCACCTCATAC |  |  |
| qNbBOP1-R | ATCGCTCGCACATACTGGACA |  |  |
| MaABI5 -SmaI-F | CCCGGGATGGTGGTTCCGGAGCCGGAAAT | | Construction of tobacco transient overexpression vector |
| MaABI5-BamHI-R | GGATCCTCACAAAGGGCAGCTCAAATTCCTT | |  |
| MaABF1 -SmaI-F | CCCGGGATGGGGTCAAATATGAACTTTAAGA | |  |
| MaABF1-BamHI-R | GGATCCTTACCAAGGGCCAGTGAGCGTCCTC | |  |
| PLGNL-F | TAGGCGTCTCGCATATCTCA | |  |
| PLGNL-R | ATATCTCCACTGACGTAAGG | |  |

Note: The underline represents the enzyme cut site, the italicized sequence Flag protein tag sequence, and the bolded part is the homology arm sequence.

**Supplementary table 2.** DGEs involved in MAPK signaling pathway in MD vs MN.

| Protein ID | Description | MD  FPKM | MD  FPKM | MD  FPKM |
| --- | --- | --- | --- | --- |
| XP_024027153.1 | Probable inactive leucine-rich repeat receptor-like protein kinase | 7.88 | 29.59 | 1.908842162 |
| XP_024024446.1 | Uncharacterized protein LOC21399766 isoform X2 | 2.266666667 | 6.323333333 | 1.480113027 |
| XP_010110030.1 | Mitogen-activated protein kinase homolog NTF3 | 24.50666667 | 50.45 | 1.041680003 |
| XP_010102289.1 | Barwin | 247.8 | 113.58 | 1.125467371 |
| XP_024032625.1 | Receptor-like protein EIX2 | 5.3 | 2.383333333 | 1.153011619 |
| XP_010100801.1 | Serine/threonine-protein kinase OXI1 | 89.90666667 | 37.82 | 1.249278734 |
| XP_010093425.1 | Ethylene-responsive transcription factor ERF096 | 193.8533333 | 79.33333333 | 1.28896647 |
| XP_010109984.1 | Calmodulin | 212.7466667 | 79.79666667 | 1.41473614 |
| XP_010103728.2 | Disease resistance protein RLM3 isoform X1 | 15.94 | 5.733333333 | 1.475205565 |
| XP_010093429.1 | Ethylene-responsive transcription factor 1B | 77.68333333 | 27.06666667 | 1.52108788 |
| XP_010100202.1 | Transcription factor MYC2 | 24.90666667 | 7.31 | 1.768588643 |
| XP_024032633.1 | Receptor-like protein Cf-9 | 8.68 | 2.486666667 | 1.803481913 |
| XP_010086565.1 | Probable WRKY transcription factor 72 | 20.19 | 3.876666667 | 2.380752315 |
| XP_024027153.1 | Probable inactive leucine-rich repeat receptor-like protein kinase | 7.88 | 29.59 | 1.908842162 |
| XP_024024446.1 | Uncharacterized protein LOC21399766 isoform X2 | 2.266666667 | 6.323333333 | 1.480113027 |

**Supplementary table 3.** DGEs involved in flavonoid biosynthesis pathway in MD vs MN.

| Protein ID | Description | MD  FPKM | MD  FPKM | MD  FPKM |
| --- | --- | --- | --- | --- |
| XP_010089186.2 | Protein SRG1, partial | 3.8 | 0.62 | 2.615659298 |
| XP_024017825.1 | Chalcone synthase 2 | 24.80333333 | 4.69 | 2.40287419 |
| XP_010095126.1 | Codeine O-demethylase | 7.836666667 | 0.333333333 | 4.555202634 |
| XP_024020876.1 | Protein DMR6-LIKE OXYGENASE 2 isoform X2 | 96.9 | 289.37 | -1.57834679 |
| XP_010097635.1 | BAHD acyltransferase BIA1 | 20.78 | 6.873333333 | 1.596113822 |
| XP_010097637.1 | Salutaridinol 7-O-acetyltransferase | 122.6233333 | 32.39333333 | 1.920464691 |
| XP_010099134.1 | Chalcone synthase | 347.4033333 | 109.56 | 1.664890426 |
| XP_010101513.2 | Chalcone-flavonone isomerase | 6.64 | 1.986666667 | 1.740833412 |
| XP_010105825.1 | Flavonoid 3'-monooxygenase | 52.77333333 | 10.26 | 2.362778381 |
| XP_010110052.1 | Bifunctional dihydroflavonol 4-reductase/flavanone 4-reductase | 75.11 | 13.77666667 | 2.446778135 |
| XP_024030364.1 | Chalcone synthase, partial | 114.9466667 | 8.35 | 3.783044622 |
| XP_010110804.1 | Leucoanthocyanidin reductase | 154.6333333 | 62.39666667 | 1.30931048 |

**Supplementary table 4.** Important genes involved in fruit abscission

| Protein ID | MD FPKM | MN FPKM | Log2FoldChange  MD vs MN | | Description |
| --- | --- | --- | --- | --- | --- |
| XP_024029824.1 | 4024.15 | 1491.57 | 1.43 | Germin-like protein subfamily 1 member 16 | |
| XP_024026818.1 | 2462.19 | 722.15 | 1.77 | *NDR1*/*HIN1*-like protein | |
| XP_010098620.1 | 701.83 | 227.33 | 1.63 | EXORDIUM | |
| XP_010101245.2 | 1527.57 | 470.26 | 1.70 | Glucan endo-1,3-beta-glucosidase | |
| XP_024017155.1 | 544.71 | 99.73 | 2.45 | *ALP1*-like | |
| XP_010092741.1 | 171.55 | 6.42 | 4.74 | *BON1*-associated protein 2 | |
| XP_024032363.1 | 774.04 | 247.77 | 1.64 | Acidic endochitinase-like | |
